# Formation of the acenaphthylene cation as a common C_2_H_2_-loss fragment in dissociative ionization of the PAH isomers anthracene and phenanthrene†

**DOI:** 10.1039/d2cp03835h

**Published:** 2022-10-27

**Authors:** Shreyak Banhatti, Daniël B. Rap, Aude Simon, Heloïse Leboucher, Gabi Wenzel, Christine Joblin, Britta Redlich, Stephan Schlemmer, Sandra Brünken

**Affiliations:** I. Physikalisches Institut, Universität zu Köln Zülpicher Str. 77 50937 Köln Germany banhatti@ph1.uni-koeln.de; Institute for Molecules and Materials, FELIX Laboratory, Radboud University Toernooiveld 7 6525 ED Nijmegen The Netherlands sandra.bruenken@ru.nl; Laboratoire de Chimie et Physique Quantiques (LCPQ), Fédération FeRMI, CNRS & Université Toulouse III – Paul Sabatier 118 Route de Narbonne 31062 Toulouse France; Center for Interstellar Catalysis (InterCat), Department of Physics and Astronomy, Aarhus University Ny Munkegade 120 8000 Aarhus C Denmark; Institut de Recherche en Astrophysique et Planétologie (IRAP), Université Toulouse III – Paul Sabatier, CNRS, CNES 9 Avenue du Colonel Roche 31028 Toulouse France

## Abstract

Polycyclic aromatic hydrocarbons (PAHs) are thought to be a major constituent of astrophysical environments, being the carriers of the ubiquitous aromatic infrared bands (AIBs) observed in the spectra of galactic and extra-galactic sources that are irradiated by ultraviolet (UV) photons. Small (2-cycles) PAHs were unambiguously detected in the TMC-1 dark cloud, showing that PAH growth pathways exist even at low temperatures. The processing of PAHs by UV photons also leads to their fragmentation, which has been recognized in recent years as an alternative route to the generally accepted bottom-up chemical pathways for the formation of complex hydrocarbons in UV-rich interstellar regions. Here we consider the C_12_H_8_^+^ ion that is formed in our experiments from the dissociative ionization of the anthracene and phenanthrene (C_14_H_10_) molecules. By employing the sensitive action spectroscopic scheme of infrared pre-dissociation (IRPD) in a cryogenic ion trap instrument coupled to the free-electron lasers at the FELIX Laboratory, we have recorded the broadband and narrow line-width gas-phase IR spectra of the fragment ions (C_12_H_8_^+^) and also the reference spectra of three low energy isomers of C_12_H_8_^+^. By comparing the experimental spectra to those obtained from quantum chemical calculations we have identified the dominant structure of the fragment ion formed in the dissociation process to be the acenaphthylene cation for both isomeric precursors. *Ab initio* molecular dynamics simulations are presented to elucidate the fragmentation process. This result reinforces the dominant role of species containing a pentagonal ring in the photochemistry of small PAHs.

## Abstract 2

This study uses cryogenic infrared spectroscopy combined with quantum-chemical and molecular dynamics calculations to elucidate the acetylene-loss fragmentation pathway in the dissociative ionization of two isomeric polycyclic aromatic hydrocarbons.

## Graphical abstract

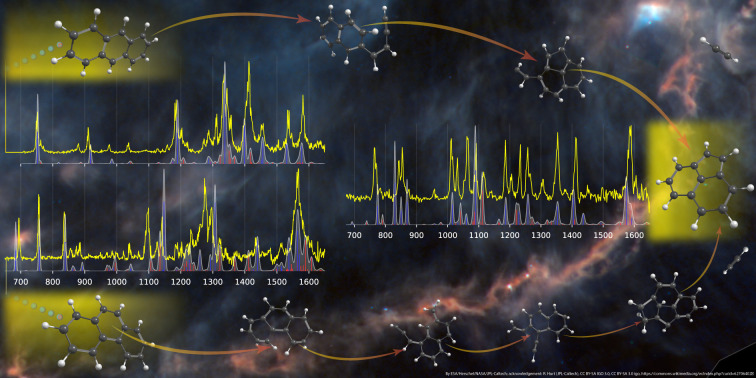

## 1 Introduction

Polycyclic aromatic hydrocarbons (PAHs) have been considered to be the carriers of the aromatic infrared bands (AIBs) for the past few decades.^1–4^ These emission bands in the 3–20 μm region observed in the spectra of many galactic and extra-galactic astronomical sources can be attributed to a large family of PAHs that absorb ultraviolet (UV) photons originating from astronomical sources and then subsequently relax *via* emission of IR photons. Models have shown that PAHs subjected to the local physical conditions of astrophysical environments can occur in different charge and hydrogenation states of varying sizes and shapes.^5–9^ Due to fragmentation processes, only large PAHs (∼50 or more carbon atoms) are expected to survive on long timescales in UV photon-rich environments.^5,7,8^

Recent radio astronomical observations have opened a new window on the study of PAHs with the identification of the small PAHs indene C_9_H_8_^10,11^ and cyanonaphthalene C_10_H_7_–CN^12^ in the dark cloud TMC-1. Small and medium-sized PAHs are also detected in primitive meteorites.^13,14^ This demonstrates the importance of small PAHs in star- and planet-forming regions and the interest to study the processes involved in their (photo)chemical evolution.

**Fig. 1:**
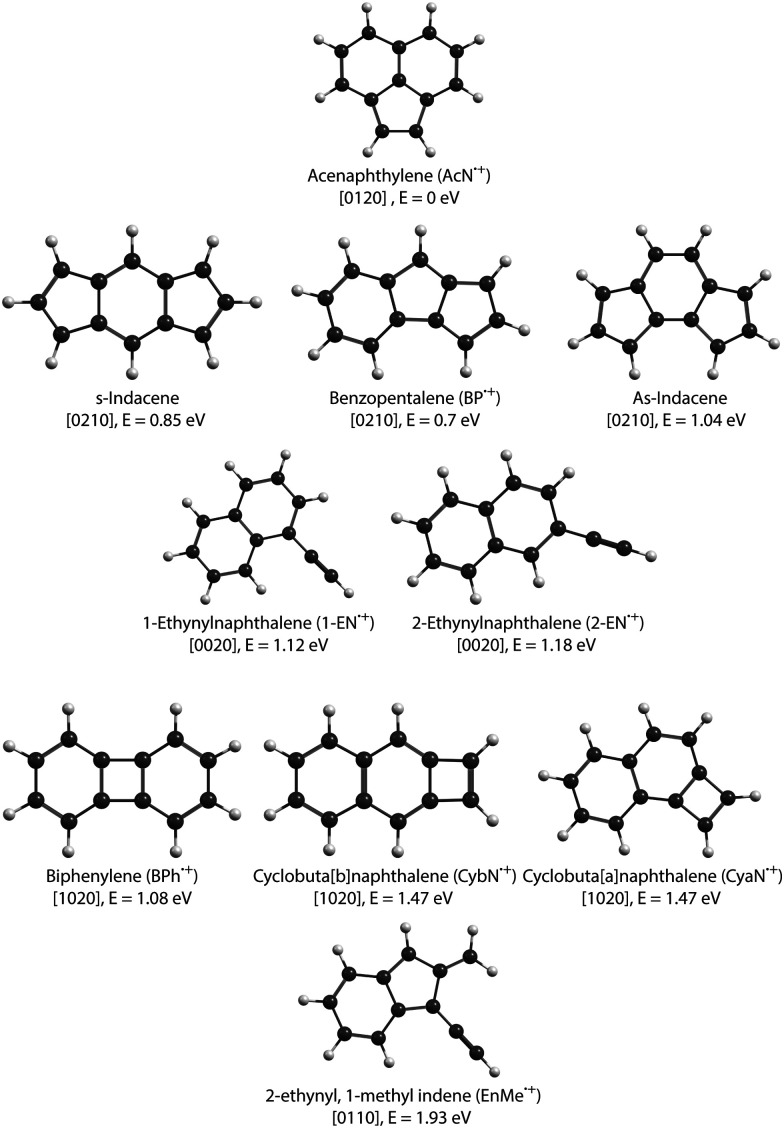
Isomers of C_12_H_8_^+^ radical cation. The electronic energies are shown relative to the lowest-energy isomer, the acenaphthylene cation, AcN˙^+^. The values noted in square brackets describe the number of *n*-carbon rings contained in each isomer with *n* = 4–7 (see Section 3.4).

In this study, we focus on the C_2_H_2_-loss fragmentation channels (leading to C_12_H_8_^+^) upon dissociative ionization of C_14_H_10_ in the case of anthracene (Anth) and its isomer phenanthrene (Phen). Previous experiments include the study of dissociative or of unimolecular dissociation, of the corresponding molecular ions Anth˙^+^ and Phen˙^+^, using vacuum UV photoionization,^15–17^ imaging photoelectron photoion coincidence spectrometry (iPEPICO) and atmospheric pressure chemical ionization – collision induced dissociation mass spectrometry (APCI-CID).^18^ Using statistical theories to analyze the data, these articles report activation energies for the main dissociation channels, *i.e.*, H, H_2_, and C_2_H_2_ loss channels. They also discuss possible structures for C_12_H_8_^+^ formed by C_2_H_2_ loss. Gotkis *et al.*^15^ proposed the acenaphthylene radical cation in the case of Phen˙^+^ dissociation but further studies by Ling *et al.*^19^ and Ling and Lifshitz^16^ favoured biphenylene for both Phen˙^+^ and Anth˙^+^. West *et al.*^18^ further discussed whether it is the biphenylene or cyclobuta[*b*]naphthalene cation (see Fig. 1 for an overview of fragment structures). Finally, Johansson *et al.*^20^ identified a possible low activation energy C_2_H_2_-dissociation channel for anthracene (C_14_H_10_) and acridine (C_13_H_9_N) cations. Their quantum chemical calculations predict three different cationic products for anthracene formed in their collision-induced dissociation study: 2-ethynylnaphthalene, biphenylene, and acenaphthylene (see Fig. 1).

Vibrational spectroscopy of molecular ions is a powerful tool for their structural and isomeric characterization. Advancements in spectroscopic techniques, especially involving the use of ion traps and powerful free-electron lasers, have enabled the development of versatile infrared (IR) action spectroscopic methods such as IR multi-photon dissociation (IRMPD)^21,22^ and IR pre-dissociation (IRPD) of complexes with rare gas atoms. In the past, these techniques have been successfully employed to elucidate the isomeric composition of cationic fragmentation products upon dissociative ionization of PAHs and related species. Using IRPD in a cryogenic ion trap, Jusko *et al.*^23^ recorded the mid-IR spectra of Ne-tagged C_7_H_7_^+^ and revealed its two isomeric forms, *i.e.*, benzylium^+^ and tropylium^+^. Ne-tagged doubly dehydrogenated pyrene (ddPy^+^) was spectroscopically investigated using the same technique by Panchagnula *et al.*^24^ The results showed that its spectral features correspond to a mixture of different isomers (4,5-ddPy^+^ and 1,2-ddPy^+^) favoured by the loss of hydrogen from the same aromatic ring. Bouwman *et al.*^25^ provided the first spectroscopic evidence, using IRMPD, for formation of the pentalene^+^ isomer in the dissociative ionization of naphthalene *via* loss of C_2_H_2_.

Following these studies, we perform here a direct spectroscopic investigation of the structure of C_12_H_8_^+^ formed by electron impact dissociative ionization of anthracene and phenanthrene. We record the mid-IR spectra of the Ne-tagged ions using IRPD action spectroscopy. The experiment is supported by detailed analysis using quantum chemical calculations which suggest the formation of mainly identical isomers for both PAHs. We also study the fragmentation process using molecular dynamics (MD) simulations with the electronic structure computed at the self-consistent charge density functional based tight binding (SCC-DFTB)^26^ method and discuss the branching ratios of the different isomers.

## 2 Methods

### 2.1 Studied species

The cationic PAH species studied in this work, *i.e.*, Anth˙^+^, Phen˙^+^ (C_14_H_10_^+^), and several C_12_H_8_^+^ isomers (see Fig. 1 for the dominant expected structures and a summary of their short names used throughout the text), were generated by electron impact ionization from gaseous PAH precursors. Table 1 lists the various PAH samples used in this study along with their purity. Anthracene and phenanthrene are canonical isomers of C_14_H_10_ with molar mass *M* = 178.23 g mol^−1^, both appearing as white crystals. 1-Ethynylnaphthalene (1-EN), 2-ethynylnaphthalene (2-EN), and acenaphthylene (AcN) are some of the isomers of C_12_H_8_ with *M* = 152.19 g mol^−1^. 1-EN is a brown liquid, 2-EN and AcN are yellowish powder. All samples were obtained from Sigma Aldrich.

**Table 1:** PAH samples used in this study

Sample	CAS-number	Purity
Anth	120-12-7	≥98%
Phen	85-01-8	98%
1-EN	5727-65-8	97%
2-EN	2949-26-0	—
AcN	208-96-8	99%

### 2.2 Experimental

In this section we briefly discuss the experimental setup, FELion, used in this study, with a more detailed account given by Jusko *et al.*^27^ FELion is a cryogenic 22-pole ion trap instrument coupled to the free-electron lasers at the FELIX (Free-Electron Laser for Infrared eXperiments) Laboratory.^28^ In this study FEL-2, covering the 600–1700 cm^−1^ range, was coupled to the FELion setup, enabling us to perform IRPD spectroscopic measurements over the whole vibrational fingerprint region. Vapours of PAHs are introduced into an electron impact (EI) ionization source at pressures of several 10^−5^ mbar. Depending on the electron energy, which is tunable between 0 to 100 eV, several singly and doubly charged parent and fragment cations can be observed as discussed in detail in our previous study.^29^ A 100 ms long pulse of the target fragment cation, *e.g.*, of mass-to-charge ratio *m*/*z* 152 corresponding to the acetylene loss fragment, C_12_H_8_^+^, is mass-selected with a quadrupole mass filter to then be cooled and stored (typically for 0.6 or 1.6 seconds) in the cryogenic 22-pole ion trap.^30^ A gas mixture of helium (He)–neon (Ne) (ratio 3 : 1) is pulsed for 100–150 ms into the trap at the beginning of the storage cycle and collisionally cools the ions close to the nominal 6.5 K of the ion trap walls. Ternary collisions lead to the formation of van der Waals complexes of the ion with one Ne atom in the trap.

The IRPD spectra are recorded by counting the number of Ne-complexed ions as a function of excitation frequency with a Daly detector.^31^ The fragment-Ne complexes dissociate due to resonant vibrational excitation while scanning the FEL-2 IR laser. The FEL-2 laser is pulsed at 10 Hz which means up to 16 macro-pulses are admitted into the trap for storage times of up to 1.6 s. Each data point at a given wavenumber is recorded under identical conditions for a minimum of three iterations and averaged. The spectrum is normalized using1
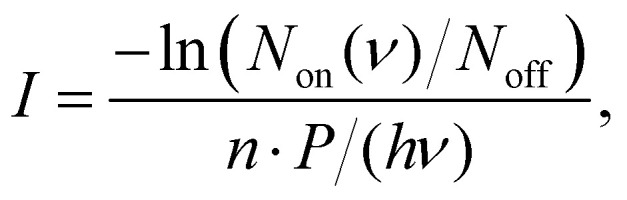
with laser pulse energy *P* (typically a few to a few ten mJ at the trap location), number of pulses *n*, number of complexed ions *N*_on_(*ν*) at a given frequency *ν*, baseline value *N*_off_; giving the intensity *I* in units of relative cross-section per photon. Multiple spectra are then averaged to obtain the final spectra shown in Section 3.2. The strong and narrow bands observed in the spectra have FWHM of less than 10 cm^−1^ on average as a consequence of the FEL-2 bandwidth (FWHM Δ*ν̃* ≈ (0.7–1.2)%·*ν̃*).

In this experiment, saturation depletion measurements^27^ provide useful information about the isomeric ratio present in the fragment-Ne complexes, a method successfully proven in Jusko *et al.*^23^ to quantify isomeric abundances of benzylium and tropylium cations, C_7_H_7_^+^. Here, the relative depletion of the fragment-Ne complexes is recorded as a function of the laser energy deposited at a wavelength corresponding to a specific vibrational band. This depletion is then compared to the ion signal observed at an off-resonance frequency to account for any losses due to non-radiative processes. The saturation depletion value then directly yields the relative abundance of the isomer that is responsible for the chosen vibrational band.

### 2.3 Theoretical

#### 2.3.1 Quantum chemical calculations

The structures, energetics and IR spectra were obtained with an electronic structure computed at the DFT level of theory using the Gaussian16 suite of programs.^32^ The theoretical IR spectra were computed in the harmonic approximation by full diagonalization of the Hessian matrix after local optimisation. Anharmonic effects were estimated using the vibrational second-order perturbation theory (VPT2) method with standard threshold settings for the treatment of resonances as implemented in the Gaussian16 suite of programs.^33^ We should note here that the VPT2 method implemented in Gaussian16 only provides a basic treatment of resonances, but that a more accurate calculation, which would require an explicit variational treatment of resonating polyads, is beyond the scope of the present work. The B3LYP hybrid functional in conjunction with the 6-311G(d,p) basis set was used, as this combination was shown to provide reliable anharmonic frequencies when using the VPT2 method.^34^ For the parent and fragment cations additional anharmonic frequency calculations were performed at the B97-1/cc-pvtz, B3LYP/Def2TZVPP, and B3LYP/N07D level of theory, but the agreement to experiment did not improve compared to the B3LYP/6-311G(dp) level of theory. A Gaussian profile with FWHM of 0.7%·*ν̃* similar to the experimentally observed FWHM is considered for convolving the quantum chemically calculated spectra throughout this study. The electronic energies and the zero-point vibrational energy corrections for the ions on the potential energy surface (PES) of the 1-EN˙^+^ to AcN˙^+^ isomerization have been calculated at the B3LYP-GD3/6-311G(d,p) level of theory. The transition states have been validated by performing intrinsic reaction coordinate (IRC) calculations. Although the presence of the Ne tag will have an effect on the vibrational band positions we expect it to be small as previously observed.^29,35–38^

#### 2.3.2 Molecular dynamics calculations

Insights into the dissociation dynamics of Anth˙^+^ and Phen˙^+^ were investigated by MD simulations using the approach developed by Simon *et al.*^39^ to study the evolution of PAHs at high internal energy and that was further applied to specific cases in comparison with experimental results.^40–42^ Briefly, the MD simulations are run in the Born–Oppenheimer approximation with the electronic structure described on-the-fly by SCC-DFTB^26^ using the modified set of C–H parameters recently published.^43^ This approach is simply referred to as MD/DFTB in the rest of the manuscript. The explicit description of electronic structure allows the description of chemical evolution such as isomerization and dissociation reactions.

In the present work, several hundreds (360 to 720) of MD/DFTB simulations of 1 ns to 2 ns duration were run for Anth˙^+^ and Phen˙^+^ (δ*t* = 0.1 fs) random initial velocities in the microcanonical (*NVE*) ensemble at 17.5, 18.5 and 20 eV. These energies were chosen as they are the lowest values for which fragmentation is observed within the nanosecond timescale. This allowed us to gain insights into reaction kinetics, branching ratios, and mechanisms. The results are described in Section 3.4. All MD/DFTB simulations were achieved using the deMonNano code.^44^

## 3 Results and discussion

### 3.1 Dissociative ionization of anthracene and phenanthrene

**Fig. 2:**
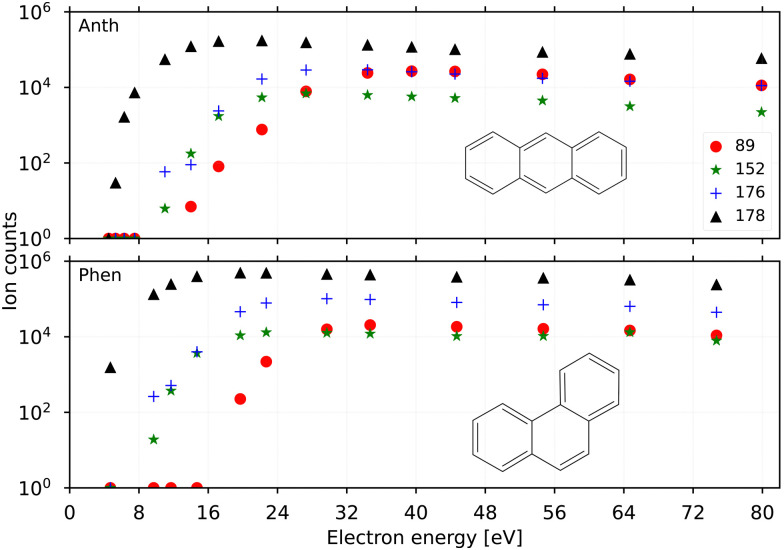
Breakdown curves for anthracene (top panel) and phenanthrene (bottom panel) showing the parent ions *m*/*z* 178, C_14_H_10_^+^ (black triangles), and their fragments *m*/*z* 89, C_14_H_10_^2+^ (red dots); *m*/*z* 152, C_12_H_8_^+^ (green stars); *m*/*z* 176, C_14_H_8_^+^ (blue crosses).

Fig. 2 shows the breakdown curves of anthracene (top panel) and phenanthrene (bottom panel) recorded for *m*/*z* 89 (C_14_H_10_^2+^), 152 (C_12_H_8_^+^), 176 (C_14_H_8_^+^), and 178 (C_14_H_10_^+^). The data was obtained by counting the number of fragment ions produced in the EI process as a function of electron energy. The appearance energy (AE) of the parent cation (C_14_H_10_^+^) for both precursors estimated from these scans is ∼7 eV‡‡We should note that the electron impact ion source used in this experiment is not ideally suited to measure appearance energies since our electron energy has a resolution of 2.7 eV, corresponding to the voltage drop across the filament. As a result the appearance energies are shifted to lower energies. close to their previously determined EI ionization energies (IE) of 7.4(8) eV.^45,46^ The IEs measured *via* photo-ionization, *i.e.*, 7.40(25) eV (anthracene) and 7.87(10) eV (phenanthrene)^16^ are also good benchmark values for the breakdown curves shown here.

The AEs for fragmentation *via* acetylene loss and hydrogen loss in low-energy electron impact experiments were found to be similar, ∼17 eV, for anthracene by Burgt and Dunne.^45^ This is also reflected in the breakdown curves recorded here for the two channels at *m*/*z* 152 and 176 which follow similar breakdown behaviour. The breakdown curves indicate a slightly lower yield of the acetylene loss (*m*/*z* 152) compared to the doubly dehydrogenated *m*/*z* 176 at higher energies (>24 eV) for both precursor PAHs. It is also evident that phenanthrene fragments form more efficiently than anthracene from a comparison of their respective yields at electron energies of 8–16 eV. This is in agreement with the finding by Burgt *et al.*^46^ who measured the AE for C_12_H_8_^+^ from phenanthrene fragmentation to be lower at ∼15.3 eV compared to ∼17 eV for anthracene. The AE for the doubly charged *m*/*z* 89 from both precursors was predicted around 20 eV in earlier studies by Banhatti *et al.*^29^ and van der Burgt and Dunne,^45^ and this value agrees with the measured second ionization potential of 11.7(1) eV for the anthracene cation,^17^ and the present results.

### 3.2 Vibrational spectra and structural analysis

**Fig. 3:**
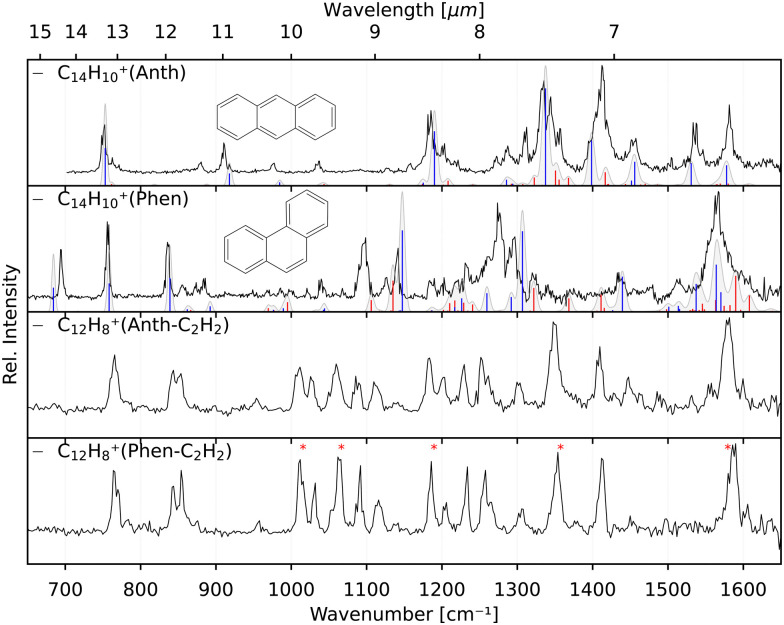
Experimental IRPD spectra of Anth˙^+^ and Phen˙^+^ and their respective C_2_H_2_ loss fragments (black). Calculated anharmonic vibrational spectra, convolved with the FEL laser linewidth, are shown for comparison for Anth˙^+^ and Phen˙^+^ including fundamental modes (blue) and combination modes (red). The spectrum of C_12_H_8_^+^(Phen–C_2_H_2_) presented in the bottom panel depicts several bands (*) that appear to have similar intensity which is caused by saturation.

In order to verify the sample purity and to investigate a possible isomerization process upon ionization,^20^ we first recorded the IRPD spectra of Anth˙^+^ and Phen˙^+^ cations (C_14_H_10_^+^, *m*/*z* 178) produced by EI (*E*_e−_ = 40 eV) of their respective vapours. The spectra are shown in the two upper panels of Fig. 3 together with their calculated anharmonic spectra at the B3LYP/6-311G(d,p) level of theory. The comparison of experiment to theory shows a good match for both isomers, with typically less than 10 cm^−1^ deviation for the fundamental modes, which are all present in the recorded IR spectra.

Previously measured IRPD spectra by Piest *et al.*^38^ of gas-phase Phen˙^+^ complexed with argon and neon also show excellent agreement with the data presented here (Fig. S1 of the ESI†), indicating a negligible effect on the vibrational frequencies between the two rare gas tags. Recent IRMPD data of Phen˙^+^ by Wiersma *et al.*^47^ in the far-infrared does not cover the same frequency region as our work. For Anth˙^+^, the data shown in Fig. 3 is the first narrow-bandwidth IRPD spectrum recorded, and it shows qualitative agreement with an earlier IRMPD spectrum by Oomens *et al.*^48^ Saturation depletion measurements at two unique band positions for Anth˙^+^ (746 cm^−1^, 1410 cm^−1^) and Phen˙^+^ (755 cm^−1^, 836 cm^−1^), respectively, prove that only one structural isomer with *m*/*z* 178, the canonical isomers, respectively, are formed in each case; *i.e.*, no isomerization, as predicted previously as a competing channel to dissociation,^16^ is observed for the direct ionization products.

**Fig. 4:**
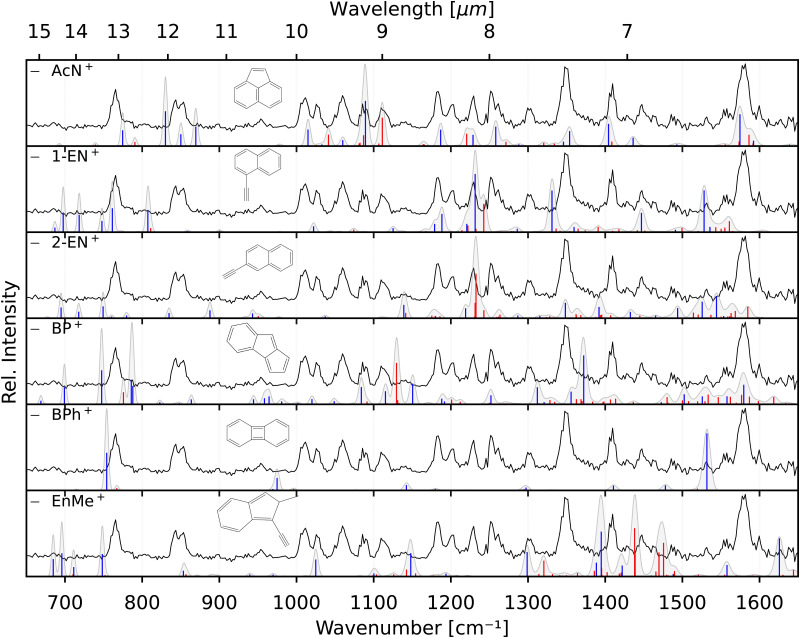
Experimental IRPD anthracene fragment spectrum (C_12_H_8_^+^, black, all panels) compared with calculated anharmonic vibrational spectra (blue – fundamentals, red – combination modes; convolved with the FEL laser linewidth) of AcN˙^+^, 1-EN˙^+^, 2-EN˙^+^, BP˙^+^, BPh˙^+^, and EnMe˙^+^.

The two lower panels of Fig. 3 show the experimental IRPD spectra of the acetylene (C_2_H_2_)-loss fragment cations (C_12_H_8_^+^, *m*/*z* 152) formed *via* dissociative EI (*E*_e−_ = 40 eV) of anthracene and phenanthrene, respectively. Even upon first visual inspection, the two spectra look almost identical, which indicates that the same fragment ion is formed from both PAH parent species. In order to elucidate the structure of the (common) fragment ion we performed quantum chemical calculations of the vibrational spectra of the lowest-energy isomers shown in Fig. 1. The calculated spectra for some of these isomers are shown in Fig. 4 and are compared to the experimental *m*/*z* 152 fragment IRPD spectrum obtained from the anthracene precursor. The calculated spectrum of acenaphthylene (AcN˙^+^) clearly shows the best match with the measured IRPD spectrum of the anthracene (and phenanthrene) fragment ion. All dominant fundamental and several combination modes of AcN˙^+^ above 1000 cm^−1^ are present in the experimental fragment spectrum. At lower frequencies the assignment is more ambiguous based on the calculated band positions.

However, saturation depletion measurements at several bands of the fragments produced from the two precursors suggest that there may be more than one isomer formed. For example, we observe greater than 94(5)% depletion at three AcN˙^+^ band positions, 1090, 1111 and 1229 cm^−1^, for the phenanthrene fragment ion, and less than 85% saturation depletion in case of the anthracene fragment for the two latter bands, and only 70% for the 1090 cm^−1^ band. This indicates that phenanthrene mainly forms AcN˙^+^, whereas anthracene, in addition to forming AcN˙^+^ as the dominant isomer with about 70% relative abundance, forms another unique fragment isomer likely among those in Fig. 1. One important aspect seen in the mass spectra (see Fig. S5 of the ESI†) of Phen and Anth is the peak at *m*/*z* 179, isotopic substitution of one ^12^C with ^13^C, with a peak intensity of 16% of *m*/*z* 178, which is approximately equal to the natural abundance of the ^13^C isotope in Phen˙^+^ and Anth˙^+^. The peak at *m*/*z* 151 indicates the presence of a C_2_H_3_ loss channel. Thus, the fragment cation formed by the loss of C_2_H_3_ and C_2_H_2_ from *m*/*z* 179 and 178, respectively, will both appear at *m*/*z* 152. As a result we expect about 10% of the *m*/*z* 152 fragment to contain other impurities, not enough to account for the only 70% depletion observed for the anthracene fragment.

As seen in Fig. 4, Anth˙^+^ shows 100% depletion at 1345 cm^−1^ which would indicate an overlapping band with the second isomer. The calculated spectra of AcN˙^+^, 1-EN˙^+^, 2-EN˙^+^, and BP˙^+^ all predict a closely lying band around 1345 cm^−1^. On the other hand, EnMe˙^+^, BPh˙^+^, and other isomers within the BP family like As- and S-indacene can be ruled out easily from the lack of overlapping bands, and the absence of the calculated strong bands, *e.g.*, at 1531 cm^−1^, in the experimental spectra (a comparison with calculated spectra of these isomers is shown in Fig. S4 of the ESI†). We also performed saturation depletion measurements at a lower electron energy (*E*_e−_ = 14.7 eV) to possibly shift the isomer ratios, as was demonstrated in our earlier study on tropylium and benzylium cations formed by EI of toluene.^23^ However, the resulting spectra did not show any noticeable change in the isomeric ratio.

To obtain additional information on the isomeric mixture we utilized Pearson's correlation coefficient^49^ and compared the anthracene fragment spectrum with the calculated spectra of the different isomers. The results shown in Fig. S2 of the ESI† confirm that AcN˙^+^ is the dominant isomer, having the highest positive correlation (0.5), followed by and 2-EN˙^+^ (0.15), BP˙^+^ (0.13), and 1-EN˙^+^ (0.04), and a negative correlation was found for the remaining isomers. Due to the precision of the calculated spectra, the identification of the second isomer remains elusive. As a reference, we also show the correlation values for Anth˙^+^ and Phen˙^+^ compared to calculated values in Fig. S2 (ESI†).

A likely candidate for a possible second isomer of the phenanthrene fragment is BP˙^+^ due to overlapping bands at 1090, 1111 and 1229 cm^−1^ which would account for the observed depletion value of >94%. However the concentration would have to be very low, of the order 0–10%, taking into consideration the isotopic contamination, signal-to-noise ratio and the depletion values >90(5)% observed for other unblended phenanthrene fragment bands.

### 3.3 Reference spectra

**Fig. 5:**
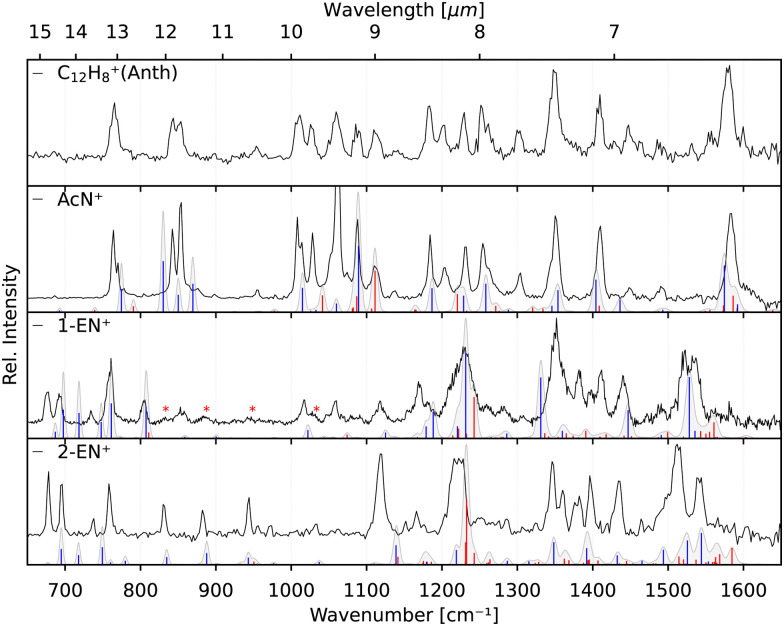
Experimental IRPD spectra of the anthracene fragment (C_12_H_8_^+^, black, top panel) and experimental IRPD reference spectra of its isomers, AcN˙^+^, 1-EN˙^+^, and 2-EN˙^+^ (black, lower three panels). Calculated anharmonic vibrational spectra, convolved with the FEL laser linewidth, are shown for comparison for the isomeric structures including fundamental modes (blue) and combination modes (red). AcN˙^+^: the band at 1062 cm^−1^ is a saturated band and out of scale on account of the normalization mentioned in Section 3.2. 1-EN˙^+^: minor contamination from 2-EN˙^+^ marked with asterisk in the 1-EN˙^+^ spectrum.

The identification of the additional isomer formed in both cases has proven to be difficult based on the calculated spectra alone. As can be seen in Fig. 4, there are shifts up to around 10 cm^−1^, and thus larger than the experimental linewidth, between the calculated and experimental band positions for the identified AcN˙^+^ isomer, in particular for the low-lying CH out-of-plane bending and CH_2_ wagging modes, for which our basic anharmonic treatment might fail.^47,50,51^ Similar effects are expected for the other candidate structures, making an assignment difficult in view of many overlapping vibrational bands. Therefore, we also recorded the reference IRPD spectra of the three most promising fragments 1-EN˙^+^, 2-EN˙^+^, and AcN˙^+^ formed by EI (*E*_e−_ = 14.7 eV) of their respective neutrals. Fig. 5 shows the fragment spectrum from the anthracene precursor together with the reference spectra of the three candidate fragment cations compared to their calculated vibrational spectra, respectively. The fragment cation spectrum presented in the top panel of Fig. 5 clearly is in large parts identical to the reference AcN˙^+^ spectrum, confirming our initial assignment. The relative intensities can vary widely between the spectra due to varying laser intensities leading to saturation of some strong bands, as seen at 1062 cm^−1^. We note in addition that strong bands below 700 cm^−1^ and between 1500–1600 cm^−1^ observed in the experimental spectra of 1-EN˙^+^ and 2-EN˙^+^ are not, or only weakly, present in the anthracene (and phenanthrene) fragment spectra. This could be due to a low abundance of specific isomers, indicating a mixture of different species, including BP˙^+^ for which we could not record a reference spectrum. The spectroscopic identification of the minor isomers in the dissociative ionization remains thus tentative.

Finally, recording the spectra of several C_12_H_8_^+^ isomers gives us the opportunity for further comparison with the anharmonic spectra at the B3LYP/6311G(d,p) level of theory. Overall, there is reasonable agreement between the measured and calculated spectra without the need of a scaling factor. We note that some bands show larger shifts such as the 1119 cm^−1^ band in 2-EN˙^+^, the 1345 cm^−1^ band in 1-EN˙^+^, and in general the bands below 900 cm^−1^. This might be due to different effects including limitations of the anharmonic calculations that provide only a very basic treatment of resonances, *e.g.*, Mulas *et al.*^52^ and a possible role of the Ne tag on the IRPD spectrum.^53^

### 3.4 Molecular dynamics simulations: dissociation pathways

**Fig. 6:**
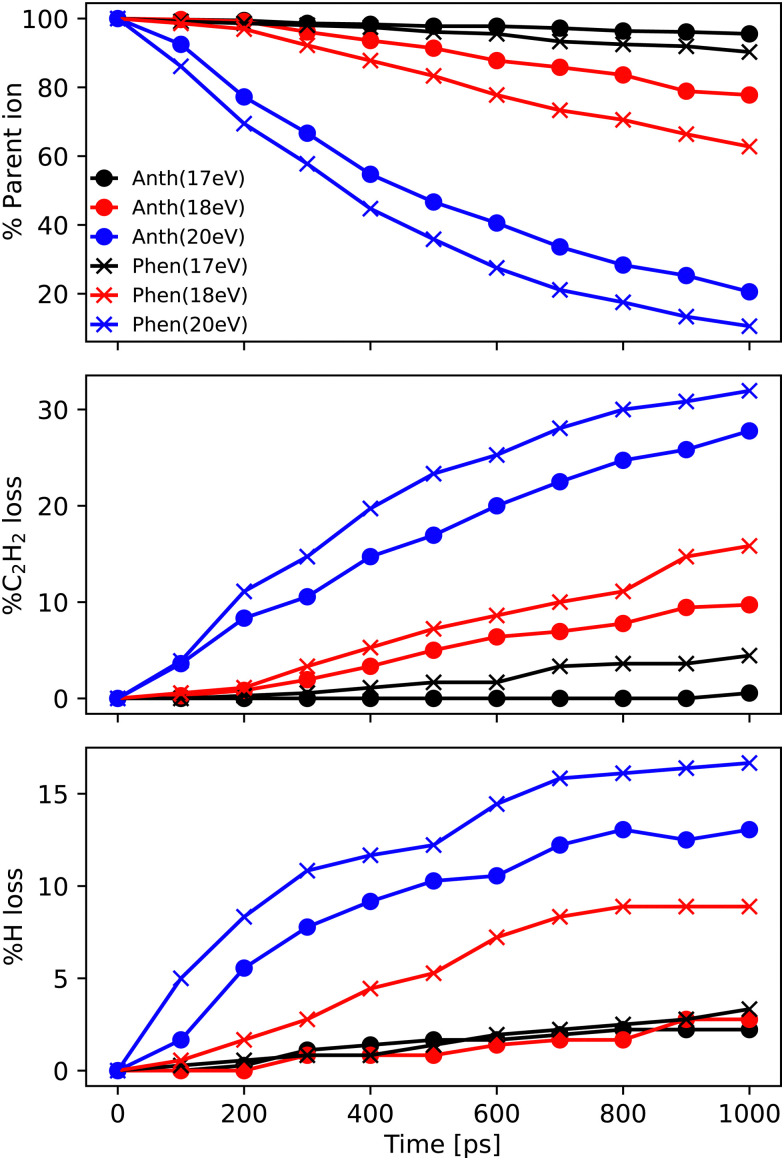
Ratio of most abundant (top panel) parent ions C_14_H_10_^+^, and fragment ions (middle panel) C_12_H_8_^+^ and (bottom panel) C_14_H_9_^+^ as a function of time during the MD/DFTB dissociation dynamics of Anth˙^+^ (dot) and Phen˙^+^ (cross) for internal energies of 17.5 eV (black), 18.5 eV (red), and 20 eV (blue). The reported data points are obtained from an average of (*NVE*) 360 simulations.

In this subsection, we report the results of the MD/DFTB simulations for Anth˙^+^ and Phen˙^+^ with internal energies of 17.5, 18.5 and 20 eV. The fraction of the parent ions as a function of time is reported in Fig. 6 (top panel). As can be seen in this figure, an energy of 17.5 eV corresponds to the lowest energy for which dissociation is observed after 1 ns. Not enough fragments are observed to deduce reliable trends for branching ratios. At 18.5 eV, the fragmentation ratio reaches 20% in the case of Anth˙^+^, and 35% in the case of Phen˙^+^. At 20 eV, the values become 80% and 90%, respectively.

At all energies, Phen˙^+^ was found to dissociate faster than Anth˙^+^ in agreement with the recorded breakdown curves. The main dissociation channel corresponds to the loss of C_2_H_2_ (see Fig. 6, middle panel) and, to a lesser extent, to the loss of H (see Fig. 6, bottom panel). As can be clearly seen in Fig. 6, fragmentation will continue after 1 ns for all energies. We chose to confirm the results and investigate the convergence of the simulations by performing additional runs at 18.5 eV of energy, with a final total of 720 simulations of 1 ns and 360 independent simulations of 2 ns (similar plots as those reported in Fig. 6 are reported in Fig. S3 of the ESI†).

Focusing on C_2_H_2_ loss, we analysed the diversity of structures for C_12_H_8_^+^ based on their number of *n*-carbon rings, *n* varying from 4 to 7. They are assigned hereafter as [*xyzt*] where *x* is the number of 4 carbon rings, *y* of 5 carbon rings, *z* of 6 carbon rings, and *t* of 7 carbon rings (see Fig. 1). The fractions corresponding to each families are reported in Table 2. At all energies the most abundant isomers correspond to [0020] (1,2-EN^+^) structures, followed by [0120] (AcN˙^+^) at 18.5 eV and 20 eV for both Phen˙^+^ and Anth˙^+^. At 17.5 eV, EnMe˙^+^ [0110] and BP˙^+^ [0210] isomers are the second most abundant isomers in the case of Anth˙^+^ and Phen˙^+^, respectively. Interestingly, regarding the [0210] structures, alternative structures to BP˙^+^ were obtained in MD simulations (see Fig. S6 of the ESI†), BP˙^+^ remaining the major one. Several [0110] isomers were also observed, differing by the lengths and positions of the linear carbon chains (see Fig. S6 of the ESI†). Several isomers were also observed in the case of the [0010] family (structures possessing a single 6 carbon ring). Regarding the [1020] family, the BPh˙^+^ isomer was observed only in one out of 360 MD simulations at 18.5 eV and only in the case of Phen˙^+^. The CyN˙^+^ was not formed in our simulations.

**Table 2:** Main isomers of C_12_H_8_^+^ formed in MD/DFTB simulations: branching ratios after 1 ns for both Phen˙^+^ and Anth˙^+^ with respect to the total number of C_12_H_8_^+^ fragments. Only ions with a branching ratio higher than 0.05 are reported. Averaging is done over 360 simulations with random velocity distributions. In the 18.5 eV case, the results of the average over 360 (2 ns) simulations and 720 (1 ns) simulations are also reported

Internal energy [eV]		17.5	18.5		20
Simulations		360	360	720	360
Fragment	Parent	1 ns	1 ns/2 ns	1 ns	1 ns
AcN	Phen˙^+^	0.12	0.41/0.35	0.33	0.31
0120	Anth˙^+^	0	0.18/0.18	0.16	0.11
BP	Phen˙^+^	0.32	0.05/0.12	0.06	0.09
0210	Anth˙^+^	0.14	0.18/0.18	0.16	0.06
1.2-EN	Phen˙^+^	0.44	0.43/0.46	0.44	0.36
0020	Anth˙^+^	0.43	0.47/0.49	0.48	0.42
EnMe	Phen˙^+^	0.12	0.05/0.15	0.11	0.17
0110	Anth˙^+^	0.43	0.18/0.15	0.16	0.27
Bz	Phen˙^+^	0	0.02/0.05	0.04	0.05
0010	Anth˙^+^	0	0/0	0.03	0.08

**Fig. 7:**
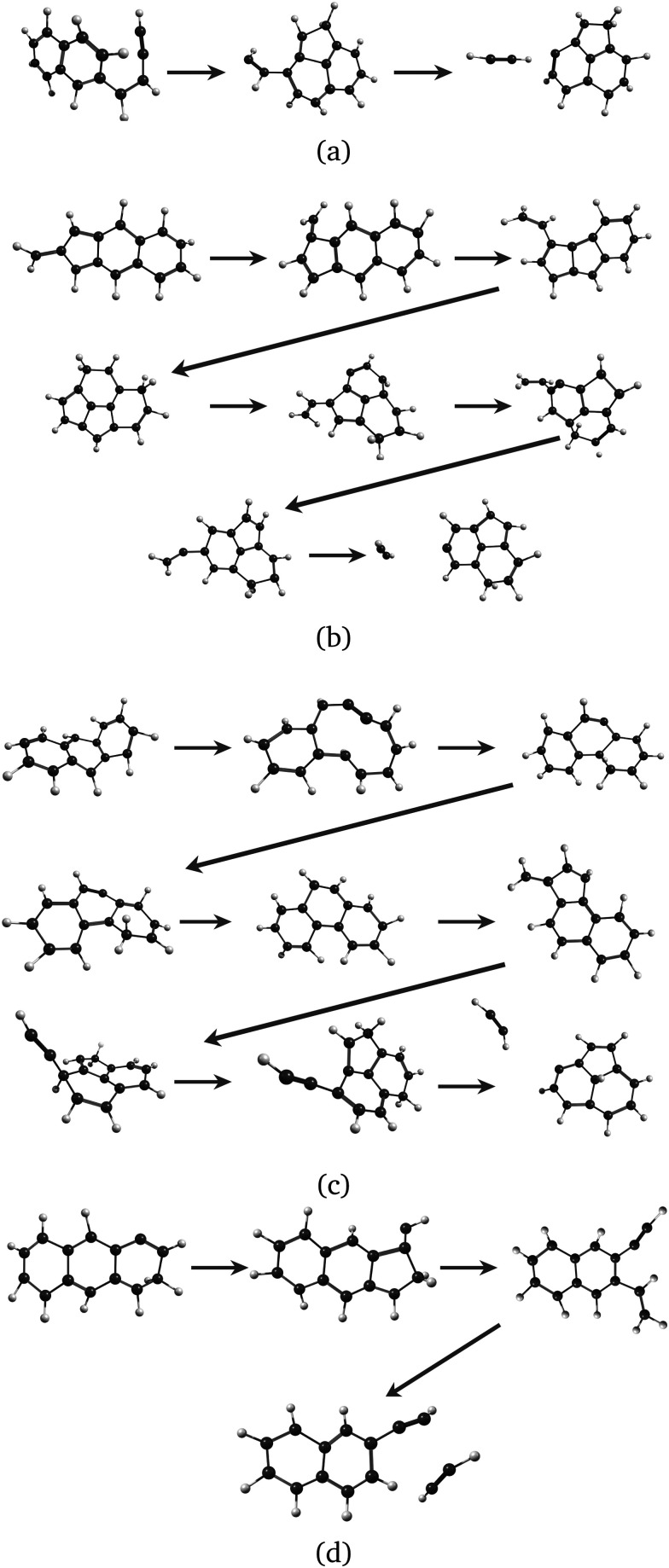
Examples of intermediates formed during the loss of C_2_H_2_ from Anth˙^+^ to lead to AcN˙^+^ (a–c) and 2-EN˙^+^ (d): snapshots withdrawn from 4 independent simulations at 18.5 eV.

Regarding the formation mechanisms, although the branching ratio (BR) for loss of C_2_H_2_ is high at 20 eV, we focus on the lower energy results (17.5 and 18.5 eV) because we expect the lower energy mechanisms to occur in experiments. In our simulations, for both Anth˙^+^ and Phen˙^+^, isomerization precedes dissociation, involving H migration, carbon skeleton rearrangement, in particular carbon ring openings and the formation of 5-carbon rings. The intermediate species often have linear carbon chains. Snapshots extracted from four MD/DFTB simulations for Anth˙^+^ at 18.5 eV are reported in Fig. 7. This illustrates three examples of mechanisms leading to AcN˙^+^, and one leading to 2-EN˙^+^. Interestingly, in Fig. 7(a) and (b), H migration will follow the loss of C_2_H_2_ to lead to AcN˙^+^. In Fig. 7(c), isomerization into Phen˙^+^ precedes dissociation. In Fig. 7(d), H migration precedes isomerization and dissociation to form 2-EN˙^+^. Examples of mechanisms leading to the formation of 2-EN˙^+^ and AcN˙^+^ from Phen˙^+^ at 17.5 eV are reported in Fig. S6 of the ESI.†

**Fig. 8:**
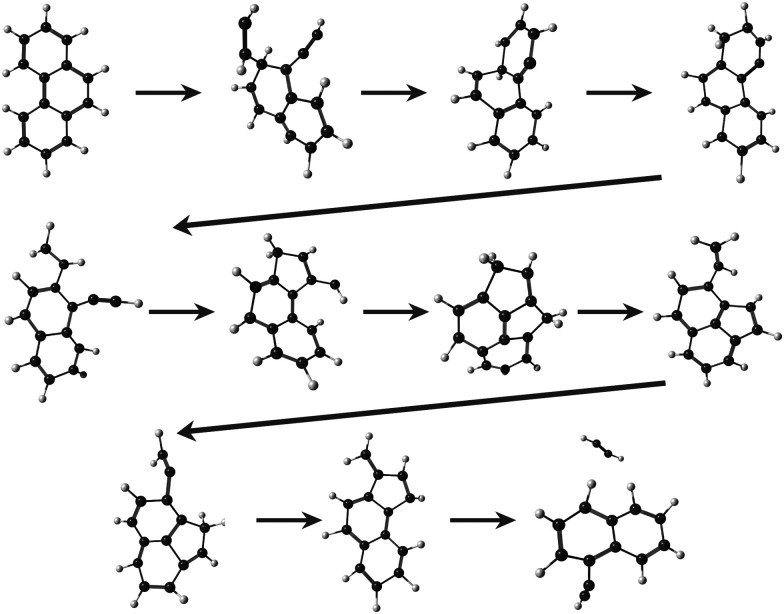
Examples of intermediates formed during the loss of C_2_H_2_ from Phen˙^+^ to lead to 1-EN˙^+^.

**Fig. 9:**
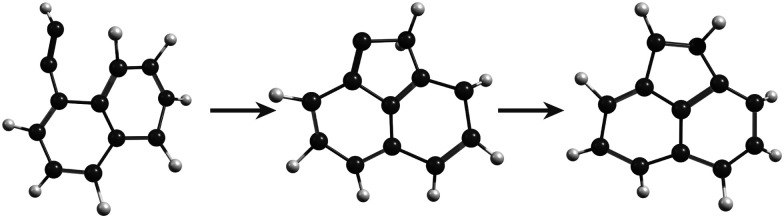
Snapshots from MD/DFTB simulations for isomerization of 1-EN˙^+^ (10 eV of internal energy) into AcN˙^+^.

In the MD/DFTB simulations, the formation of 1,2-EN^+^ isomers is in competition with AcN˙^+^ as the major fragment, contrary to our experiments. In order to account for this discrepancy, we suggest that, with an energy of at least 17.5 eV as used in the MD simulations, all energy barriers within the paths leading to 1-EN˙^+^ and 2-EN˙^+^ can be overcome. In addition to the pathway from Anth˙^+^ to 2-EN˙^+^ shown in Fig. 7(d), an example of a mechanism leading to 1-EN˙^+^ from Phen˙^+^ at 17.5 eV is depicted in Fig. 8. It involves H migration, external ring opening, and formation of a “vinylidene” (fulvene-like) structure prior to the loss of C_2_H_2_. From such a structure, it should be possible to observe isomerization to AcN˙^+^ for sufficiently long simulations or sufficient internal energy in the 1,2-EN^+^ isomer. MD simulations from 1-EN˙^+^ with 10 eV of internal energy supported the latter scenario (Fig. 9). Indeed, we observed isomerization into AcN˙^+^ following the mechanisms reported in Fig. 7, involving C–C coupling and H migration.

We should note here that in the experiment internal energies up to 30 eV might be deposited in the parent cations upon electron impact ionization. As explained in Section 2.3.2, the MD energies were chosen so as to be the lowest ones to observe the C_2_H_2_ loss, and they are not expected to mimic the experimental conditions. Furthermore, analysis of the dissociation paths in reasonable computational time is only possible at the nanosecond timescale although fragment structures are probed after several microseconds in the experiments.

### 3.5 Isomerization processes between 1-EN˙^+^ and AcN˙^+^

**Fig. 10:**
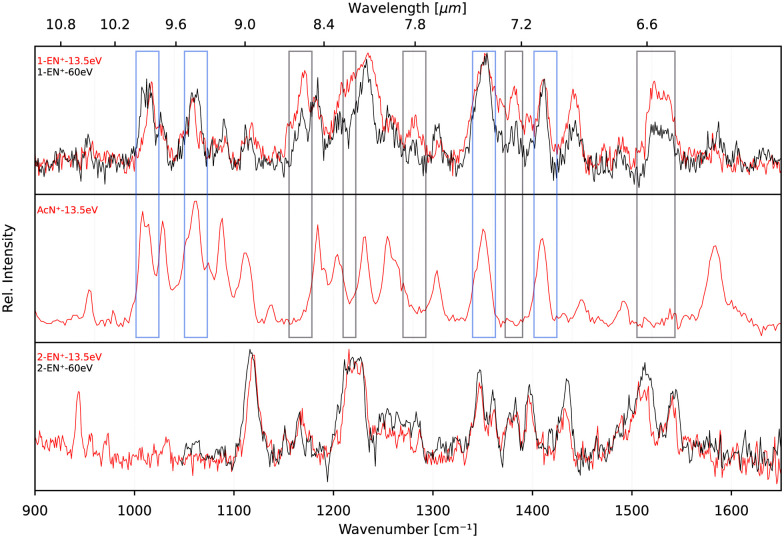
Experimental IRPD spectra of 1-EN˙^+^, ACN˙^+^, and 2-EN˙^+^ at electron impact ionization energies of 15 eV (red traces) and 60 eV (black traces). Regions where AcN˙^+^ bands overlap with 1-EN˙^+^ bands are indicated by cyan boxes, whereas areas where no AcN˙^+^ bands are present are marked with gray boxes.

In order to elucidate a potential isomerization process between the functionalized bicyclic PAH 1-EN˙^+^ and the tricyclic AcN˙^+^, we performed additional experiments where the 1-EN and 2-EN neutral precursor were subjected to varying EI energies, thereby inducing possible additional isomerization and fragmentation processes. By measuring the IR fingerprint spectrum at different electron energies, isomerization processes within the mass channel can be identified. The experimental IRPD spectra of 1-EN˙^+^ and 2-EN˙^+^ at electron energies of 15 eV and 60 eV are shown in Fig. 10. No spectral variation with EI energy was observed for 2-EN˙^+^, but significant differences in several spectral features were observed for 1-EN˙^+^. In regions marked with gray boxes, we observe a decrease in the peak intensities at higher electron energy while regions marked with cyan boxes showed no change in peak intensities. The former regions contain rather unblended bands of 1-EN˙^+^, whereas the latter regions contain many overlapping bands of AcN˙^+^, indicating that 1-EN˙^+^ isomerizes into AcN˙^+^. This opens up the possibility of a sequential process involving 1-EN˙^+^ as an intermediate before forming AcN˙^+^, in the dissociative ionization of Anth˙^+^ and Phen˙^+^, as discussed in Section 3.4.

**Fig. 11:**
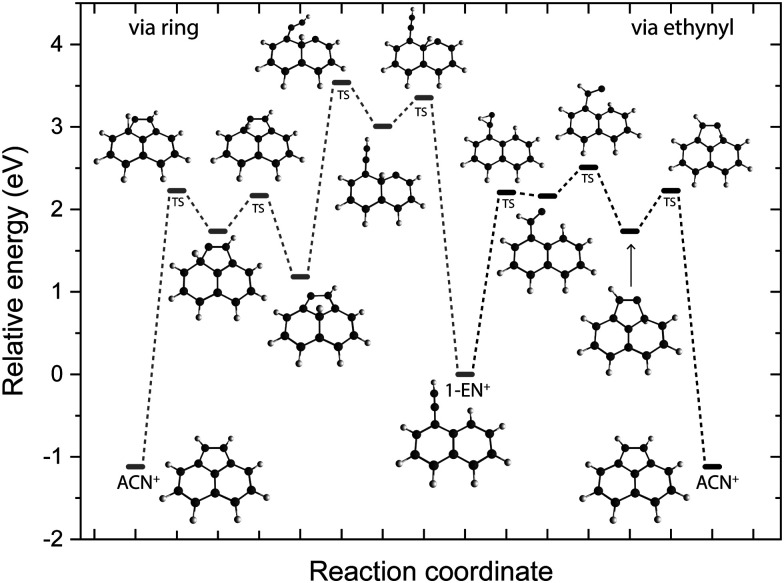
Potential energy surface of the isomerization pathways between 1-EN˙^+^ and ACN˙^+^ calculated at the B3LYP-GD3/6-311G(d,p) level of theory. The electronic energies including the zero-point vibrational energy correction are shown with respect to the 1-EN˙^+^.

To look deeper into the feasibility of an isomerization pathway for 1-EN˙^+^, we have constructed the PES of two pathways that lead to the formation of ACN˙^+^ (see Fig. 11). One isomerization pathway where the first H migration takes place on the ethynyl group has been described previously.^54^ The overall isomerization process to form ACN˙^+^ is determined to be exothermic. However, a few barriers above the entrance energy exist. The highest transition state involving the ring closing reaction is calculated to be 2.5 eV higher in energy. A second pathway is found where a hydrogen atom moves from the ring to form a sp^3^ hybridized atom in the middle of the PAH base. This process requires one more transition state and contains an even higher lying transition state at 3.5 eV.

## 4 Astronomical implications

The formation of isomer fragments or products containing pentagonal rings such as AcN˙^+^ observed in this study has been previously discussed extensively in the literature. Examples include the C_2_H_2_ loss in the dissociative ionization of naphthalene, where the pentalene cation is formed^25^ and in that of two three-cyclic N-containing polyaromatics.^55^ Petrignani *et al.*^56^ also reported the formation of the fluorenyl cation C_13_H_9_^+^ by dissociative ionization of fluorene (C_13_H_10_), 9,10-dihydrophenanthrene (C_14_H_12_), and 9,10-dihydroanthracene (C_14_H_12_). The reaction of neutral allyl radicals (C_3_H_5_˙) with acetylene (C_2_H_2_) and *o*-benzyne has been shown to lead to the formation of cyclopentadiene (C_5_H_6_) and indene, respectively, as the primary isomer products,^57,58^ and a variety of five-membered ring structures were spectroscopically detected in benzene and naphthalene discharges.^59–61^

We also note that in the AcN˙^+^ and BP˙^+^ spectra the bands around 1000 to 1150 cm^−1^ (8.7–10 μm) correspond to the in-plane C–H bending vibration involving the pentagonal ring. Features in this range were also observed in the IR spectra of rubicene˙^+^,^62^ corannulene˙^+^,^63^ and diindenoperylene˙^+^ ^64^ and were found to involve the pentagonal structures. At the era of the James Webb Space Telescope, these spectral features can serve as probes for detecting pentagonal carbon-ring containing PAHs in the interstellar medium (ISM).

## 5 Conclusions

We studied the dissociative ionization of anthracene and phenanthrene by spectroscopic analysis in the IR fingerprint region of its monocations and fragments formed *via* C_2_H_2_-loss. MD/DFTB simulations for Anth˙^+^ and Phen˙^+^ at different internal energies show that the main dissociation channel corresponds to the loss of C_2_H_2_ followed by the H-loss channel. The structural analysis of C_2_H_2_ loss fragments reveals several possible isomers with varying carbon rings (*n* = 4–7) and aliphatic chains (see Fig. 1). Strong evidence for the formation of a common product isomer AcN˙^+^ in the dissociation of Anth˙^+^ and Phen˙^+^ is shown by comparison of the recorded fragment vibrational spectra with anharmonic DFT calculations and recorded reference spectra. These pentagonal carbon-ring containing PAHs might be abundant in the ISM, and the present work provides laboratory reference data for their astronomical detection.

Anth˙^+^ fragmentation also leads to the formation of a second isomer. Based on a comparison with anharmonic DFT calculations and reference spectra, in combination with the results of MD/DFTB calculations, we suggest the ethynyl-substituted naphthalene 2-EN˙^+^ as the second isomer for this C_12_H_8_^+^ fragment since it only involves hydrogen migration and bond breaking and no geometry rearrangements as seen in Fig. 7(d). The recent discovery of two isomers of ethynyl cyclopentadiene in TMC-1^10^ suggests that PAHs containing ethynyl-chains, such as 1-EN and 2-EN, are potentially important fragments in the ISM. The presented IR spectra and calculations also revealed that 1-EN˙^+^ isomerizes into AcN˙^+^ with increasing electron energy, which might explain why phenanthrene dominantly fragments to AcN˙^+^ during dissociative ionization, with only minor contribution of another isomer. Through a combination of DFT calculations and experimental data we have also confirmed that anthracene does not isomerize to form a stable phenanthrene product upon ionization.

The results presented here demonstrate that high resolution action spectroscopy using rare-gas-tagging as implemented at the cryogenic ion trap user station FELion at FELIX, combined with sophisticated theoretical methods such as MD/DFTB, provides a powerful tool to investigate isomer selective fragmentation pathways of molecular ions such as the polycyclic aromatic hydrocarbons studied here.

## Data availability

Source data for figures in this article is available at https://doi.org/10.34973/18wv-t810. Additional data supporting this article is available in the ESI.†

## Conflicts of interest

There are no conflicts to declare.

## Supplementary Material

CP-024-D2CP03835H-s001
