# Supplementary material for: Formation of the acenaphthylene cation as a common C2H2-loss fragment in dissociative ionization of the PAH isomers anthracene and phenanthrene
Source: Phys Chem Chem Phys. 2022 Oct 27;24(44):27343–54. doi: 10.1039/d2cp03835h (PMC9673687; doi:10.1039/d2cp03835h)
Supplement: CP-024-D2CP03835H-s001 [file CP-024-D2CP03835H-s001.pdf]

Electronic Supplementary Material (ESI) for Physical Chemistry Chemical Physics.
This journal is © the Owner Societies 2022

Supplementary Information

Formation of the acenaphthylene cation as a common C_2H_2-loss fragment in dissociative ionization of the PAH isomers anthracene and phenanthrene

Shreyak Banhatti*[a], Daniël B. Rap[b], Aude Simon[c], Heloïse Leboucher[c], Gabi Wenzel[d], Christine Joblin[e], Britta Redlich[b], Stephan Schlemmer[a], Sandra Brünken*[b]

[a] *I. Physikalisches Institut, Universität zu Köln, Zülpicher Str. 77, 50937 Köln, Germany*
* *E-mail: banhatti@ph1.uni-koeln.de*

[b] *Radboud University, Institute for Molecules and Materials, FELIX Laboratory, Toernooiveld 7, 6525 ED, Nijmegen, The Netherlands*
* *E-mail: sandra.bruenken@ru.nl*

[c] *Laboratoire de Chimie et Physique Quantiques (LCPQ), Fédération FeRMI, CNRS & Université Toulouse III - Paul Sabatier, 118 Route de Narbonne, 31062 Toulouse, France*

[d] *Center for Interstellar Catalysis (InterCat), Department of Physics and Astronomy, Aarhus University, Ny Munkegade 120, 8000 Aarhus C, Denmark*

[e] *Institut de Recherche en Astrophysique et Planétologie (IRAP), Université Toulouse III - Paul Sabatier, CNRS, CNES, 9 Avenue du Colonel Roche, 31028 Toulouse, France*

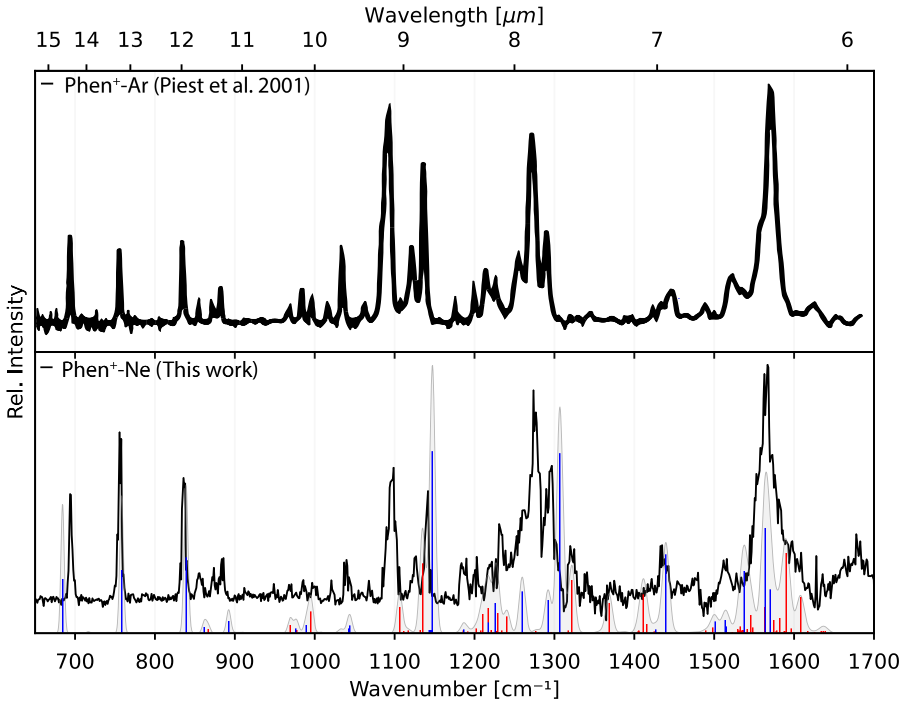

Fig. S1 Experimental infrared spectra of this work (bottom) compared with the results of molecular beam experiments by Piest et al.[1](top). The infrared predissociation spectra of $Phen^+$ clustered with neon and argon, respectively are recorded via induced cluster dissociation. Only negligible frequency shifts are observed between the two spectra. Piest et al. also recorded their electronic spectra using the same technique and expects a weaker interaction between the neon atom and the phenanthrene chromophore in the ionic groundstate w.r.t argon. The weaker interaction also indicates that a $Phen^+$ -Ne infrared spectrum should resemble the spectrum of bare cationic phenanthrene better.

1 Hans Piest et al. "Vibrational spectroscopy of gas-phase neutral and cationic phenanthrene in their electronic groundstates". In: Spectrochim. Acta A Mol. Biomol. Spectrosc. 57.4 (2001), pp. 717 –735. ISSN: 1386-1425. DOI: https://doi.org/10.1016/S1386-1425(00)00439-X. URL: http://www.sciencedirect.com/science/article/pii/S138614250000439X.

2 **https://numpy.org/doc/stable/reference/generated/numpy.corrcoef.html**.

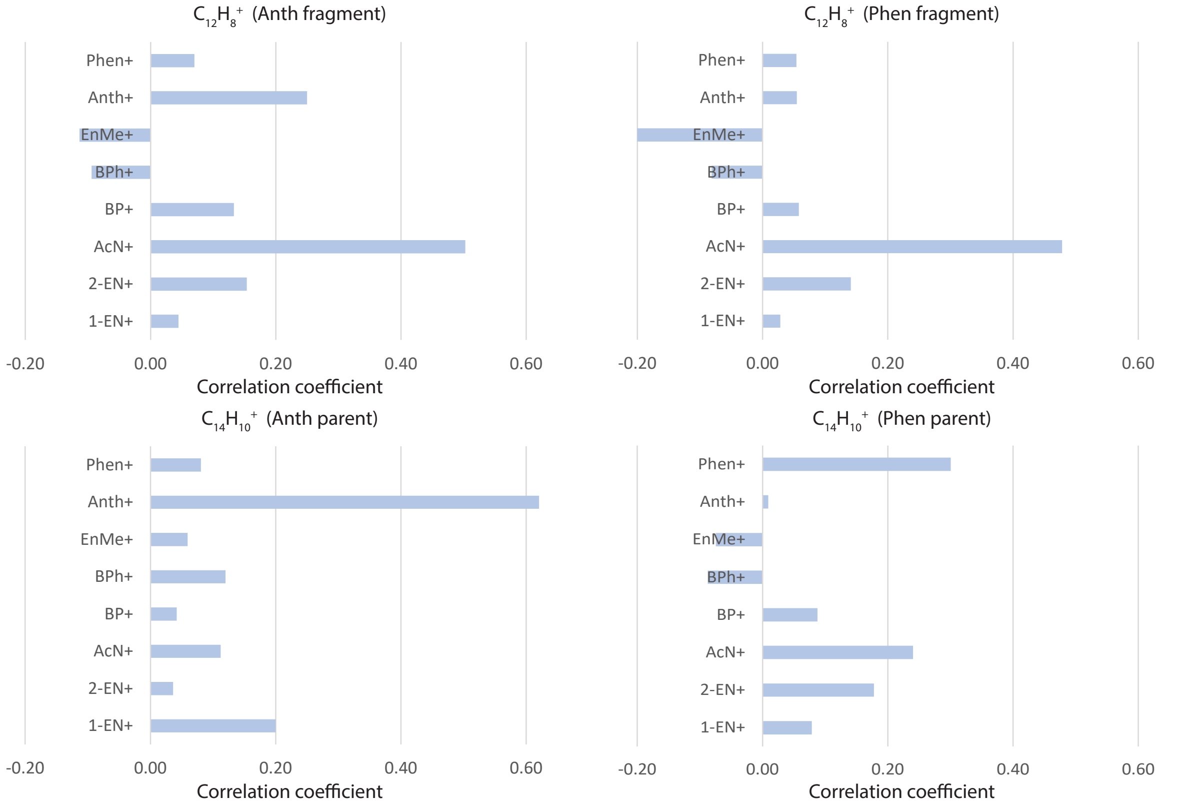

Fig. S2 Pearson's correlation coefficients calculated for experimental IRPD vibrational spectra correlated with DFT calculated (B3LYP/6-311G(d,p)) spectra of parent ions and their fragment isomers. Top left: $C_{12}H_8^+$ (Anth) fragment; Top right: $C_{12}H_8^+$ (Phen) fragment; Bottom left: $C_{14}H_{10}^+$ $Anth^+$; Bottom right: $C_{14}H_{10}^+$ $Phen^+$. Both the recorded parent cation spectra have the highest correlation with their respective calculated spectra. The comparison with the other fragment isomers is simply shown to illustrate the method. In case of $Phen^+$ parent we observe a correlation of only 0.3 compared to 0.62 for $Anth^+$ parent, which is mainly due to the fact that our experimental data for phenanthrene was noisy compared to anthracene, especially around 1300 cm^{-1} . The correlation is calculated between $900-1650$ cm^{-1} using the experimental and calculated intensities for each wavenumber point (the latter are folded with the laser linewidth). A value of +1 of correlation implies a perfect match between the recorded and the calculated spectra. A value of or below 0 implies that there is no linear dependency between the spectra. The Pearson's correlation coefficients were calculated using python as implemented in its numpy package[2].

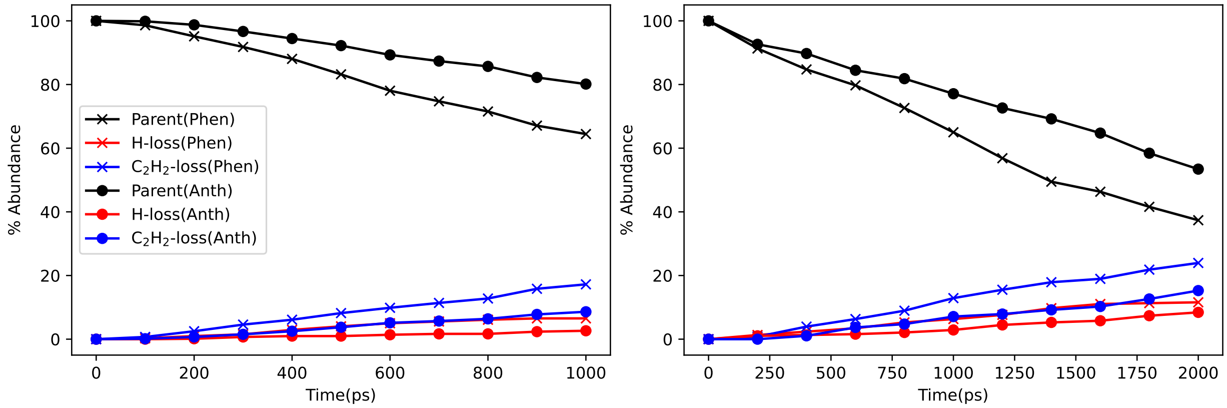

Fig. S3 Results of molecular dynamics simulations of the unimolecular fragmentation of $Phen^+$ (crosses) and $Anth^+$ (dots): Left: 720 simulations over 1 ns. Right: 360 simulations over 2 ns. The relative yield of the parent ions $C_{14}H_{10}^+$ (black), and most abundant fragment ions $C_{12}H_8^+$ (blue) and $C_{14}H_9^+$ (red) as a function of time during the MD/DFTB dissociation dynamics are plotted for internal energies of 18.5 eV.

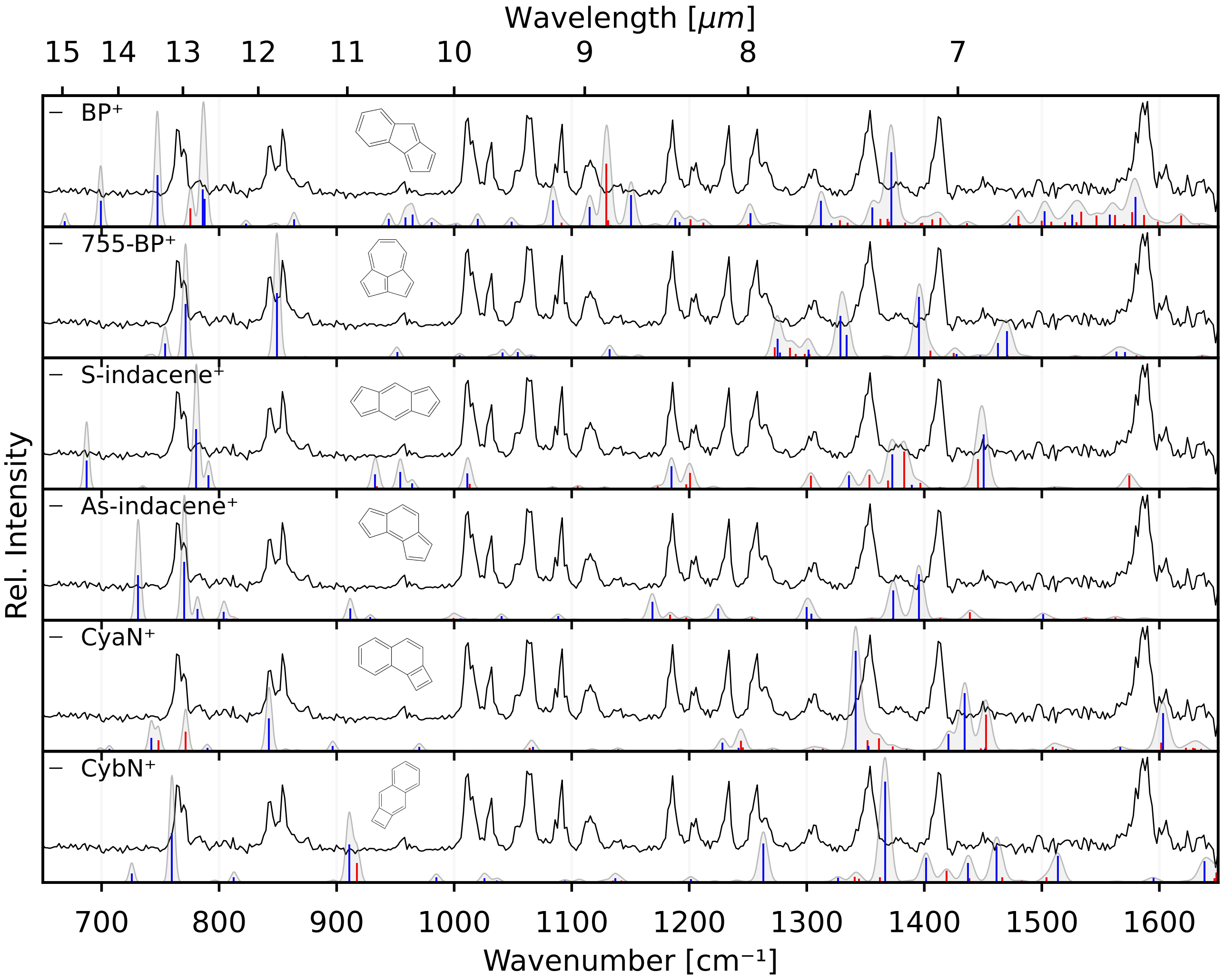

Fig. S4 Experimental IRPD spectrum (black) of the C_2H_2-loss fragment ion produced from dissociative ionization of Phencompared with anharmonic DFT calculations (B3LYP-GD3/6-311G(d,p)) of BP^+, 755-BP^+, S-indacene$^+$, As-indacene$^+$, Cy[a]N^+ and Cy[b]N^+. (blue - fundamentals, red - combination modes; convolved with the FEL laser linewidth).

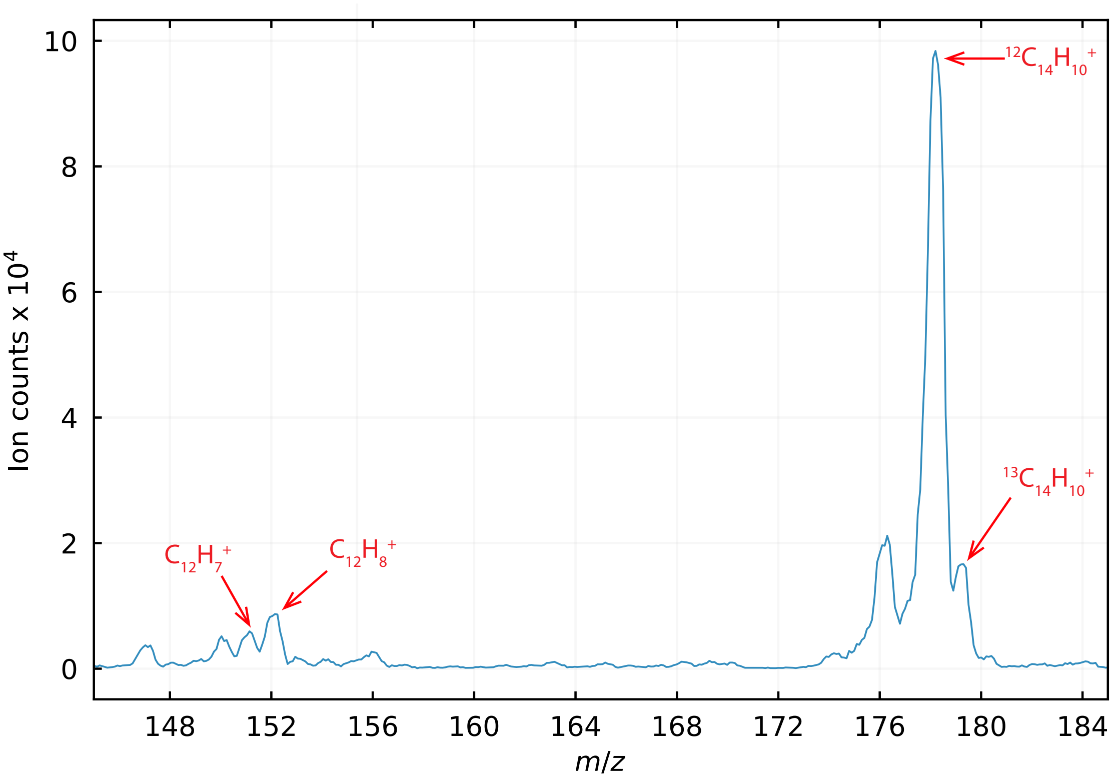

Fig. S5 Mass spectrum of anthracene recorded at an ionization energy of 40 eV. m/z 179 has $\approx 16\%$ abundance relative to m/z 178 which is similar to the expected abundance assuming that one of the 14 carbon atoms is replaced by its ^{13}C isotope in natural abundance. Peaks observed at m/z 150, 151, 152, and 176 correspond dominantly to fragments formed via loss of, -C_2H_4, -C_2H_3, -C_2H_2, and -H_2, respectively, but can contain similar abundances of ^{13}C substituted species.

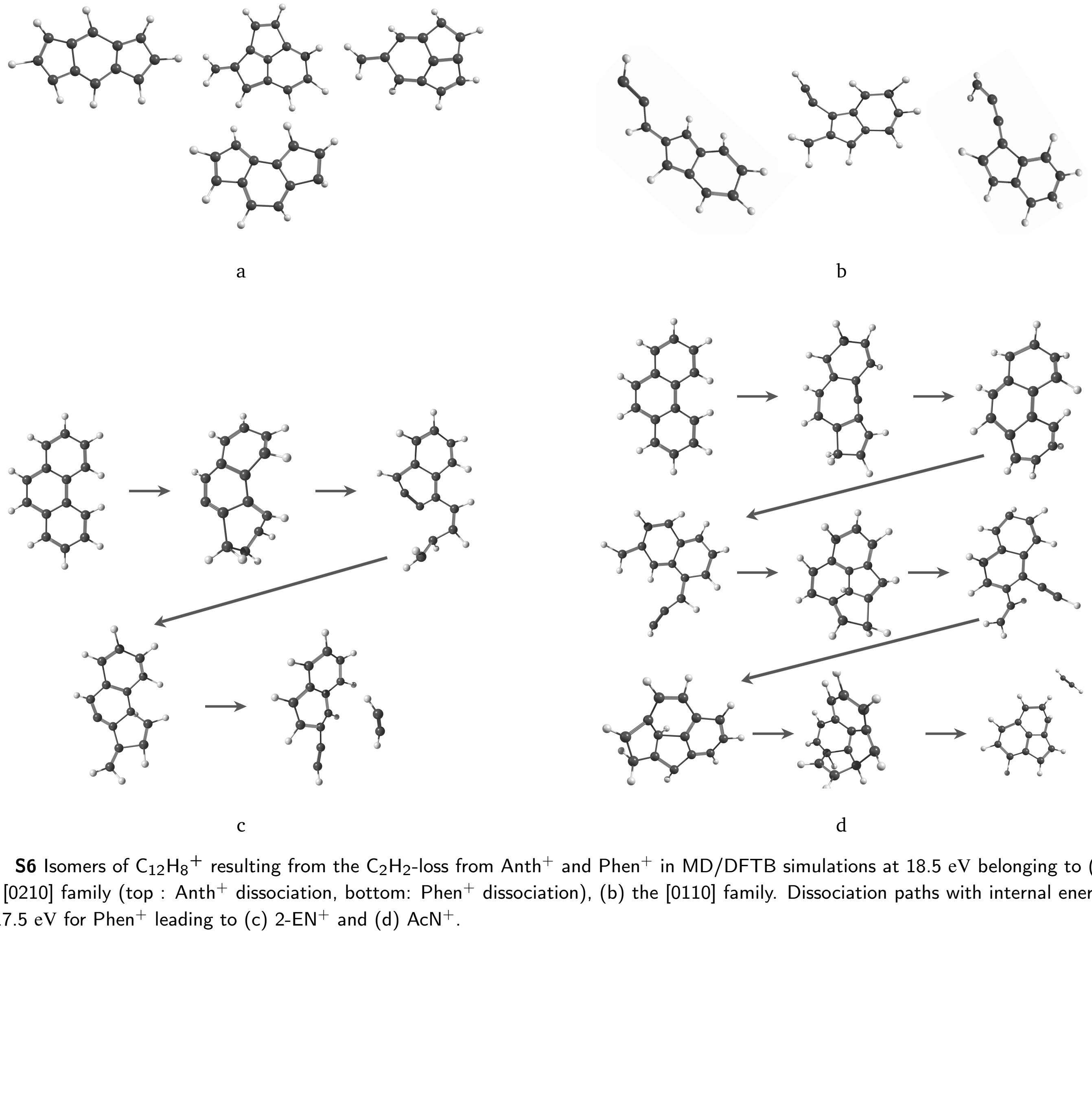

Fig. S6 Isomers of $C_{12}H_8{}^+$ resulting from the C_2H_2-loss from $Anth^+$ and $Phen^+$ in MD/DFTB simulations at 18.5 eV belonging to (a) the [0210] family (top : $Anth^+$ dissociation, bottom: $Phen^+$ dissociation), (b) the [0110] family. Dissociation paths with internal energy of 17.5 eV for $Phen^+$ leading to (c) 2-EN^+ and (d) AcN^+.
